# Movement behaviors and cardiorespiratory fitness – a cross-sectional compositional data analysis among German adults

**DOI:** 10.1186/s13102-025-01112-7

**Published:** 2025-03-28

**Authors:** Alexander Roosz, Martin Bahls, Sabina Ulbricht, Antje Ullrich, Anne Obst, Beate Stubbe, Ralf Ewert, Sabine Kaczmarek, Till Ittermann, Marcus Dörr, Lisa Voigt

**Affiliations:** 1 Department of Internal Medicine B, University Medicine Greifswald, Ferdinand-Sauerbruch-Str, 17475 Greifswald, Germany; 2 German Centre for Cardiovascular Research (DZHK), Partner Site Greifswald, Greifswald, Germany; 3 Community Medicine Greifswald, SHIP-KEF, University Medicine Greifswald, 17475 Greifswald, Germany

**Keywords:** Accelerometry, Cardiopulmonary exercise testing, Physical activity, Stationary time, Sex differences

## Abstract

**Background:**

We investigated associations of movement behaviors (moderate-vigorous physical activity, light physical activity, and stationary time) with various parameters measured during cardiopulmonary exercise testing. We applied compositional data analysis to account for the relative contributions of different movement behaviors to the overall time budget of the waking day.

**Methods:**

We used data from 1,396 participants of the cross-sectional population-based Study of Health in Pomerania (SHIP-TREND-1), who provided valid accelerometer data worn on the hip for seven days during waking hours and participated in cardiopulmonary exercise testing on a cycle ergometer (*n* = 1,396 participants with a mean age of 57.1 (SD 13.2, 51% men). Linear regression models applying compositional data analysis were used to examine associations of proportions of movement behaviors (exposure) with parameters derived during cardiopulmonary exercise testing (outcome) normalized for body weight and stratified by sex. Models were adjusted for age, education, smoking, and partnership, except the %predicted VO_2_peak model, where age was omitted, as it is part of the calculation of the %predicted VO_2_peak. In models examining O_2_pulse or HRmax, individuals using beta blockers were excluded.

**Results:**

In males and females, more time spent in moderate-to-vigorous physical activity was associated with greater VO_2_VT1, VO_2_peak, and VO_2_ recovery after 60 s (all *p* < 0.01). Greater moderate-to-vigorous physical activity was also related to higher %predicted VO_2_peak and maximum heart rate in males and to higher VO_2_/work in females (all *p* < 0.01). In both sexes, more time in stationary time was associated with less %predicted VO_2_peak (*p* < 0.01). More light intensity physical activity was associated to higher %predicted VO_2_peak in both sexes and with lower VO_2_/work in women (all *p* < 0.01). Greater stationary time was related to less VO_2_/work, VO_2_VT1, and VO_2_peak in males and to less VO_2_ recovery after 60 s and O_2_pulse in females (*p* values < 0.05).

**Conclusion:**

Moderate-to-vigorous physical activity (positive) and stationary time (inverse) influence parameters derived during cardiopulmonary exercise testing irrespective of age, smoking, and living in a relationship. The sex specific effects were rather small. Hence, promoting physical activity should be encouraged to increase cardiorespiratory fitness.

**Graphical Abstract:**

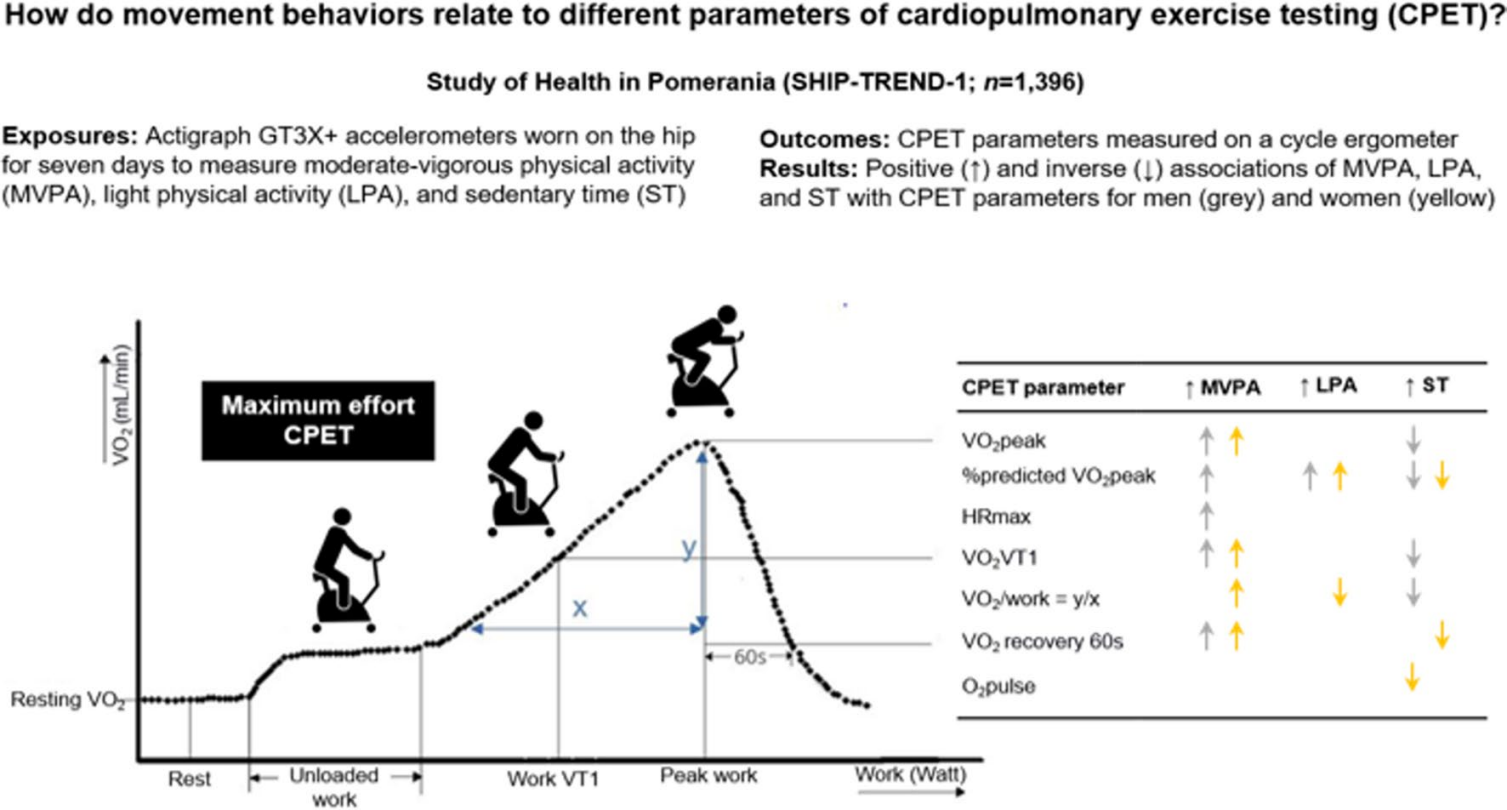

**Supplementary Information:**

The online version contains supplementary material available at 10.1186/s13102-025-01112-7.

## Introduction

Physical activity is the hallmark of an active lifestyle [1]. However, more than one-third of the European adult population is physically inactive [2]. The lack of physical activity has severe consequences for individuals as well as societal health care systems, considering that physical inactivity is a risk factor for most noncommunicable diseases [3–5] and increases premature mortality up to 30% [6]. Estimations show that up to 3.9 million premature deaths per year can be avoided through physical activity [7]. According to the World Health Organization, adults should achieve 150–300 min of moderate-intensity physical activity, 75–150 min of vigorous-intensity physical activity, or some equivalent combination of moderate-to-vigorous-intensity aerobic physical activity per week [8]. Hence, stationary time should be reduced to counteract adverse health consequences independent of low physical activity [9].

Scientist encourage that cardiorespiratory fitness is a vital clinical sign determined by cardiopulmonary exercise testing [10, 11]. High cardiorespiratory fitness (i.e., maximal oxygen consumption, VO_2_peak) is inversely associated with all-cause, cardiovascular and cancer mortality [12]. Cardiorespiratory fitness is also strongly associated with established risk factors such as hypertension, high levels of low-density lipoprotein cholesterol and type 2 diabetes mellitus [13, 14]. A meta-analysis revealed that a 1-metabolic equivalent of task (MET) greater cardiorespiratory fitness was associated with a 13% and 15% lower risk of all-cause and cardiovascular mortality, respectively [15]. In addition to VO_2_peak, other parameters, such as VO_2_ at the ventilatory threshold (VO_2_VT1), can be measured during different phases of the cardiopulmonary exercise test. Information from these values may lead to a more comprehensive view of the systemic response to cardiopulmonary exercise testing [16, 17].

Physical activity and cardiorespiratory fitness are cardioprotective measures and are sometimes considered surrogates. However, both are independently associated with lower cardiovascular risk, and both should be considered when estimating risk for cardiovascular diseases [18]. Interestingly, cardiorespiratory fitness is influenced by a large number of correlates and up to 50% by genetics [19–21]. In addition, moderate vigorous physical activity explains only a very small proportion of the variance in cardiorespiratory fitness [22, 23]. While there are hints for a partial connection, their relationship is still unclear [24].

Previous studies on cardiorespiratory fitness have analyzed different forms of physical activity, e.g., moderate-vigorous and light physical activity, and stationary time, with a focus on their independent effects on health outcomes, ignoring their inherent compositional nature. As the day is limited to 24 h, there is a degree of codependency between these behaviors. Specifically, if there is an increase in the amount of time per day spent in one behavior, then one or more of the other behaviors have to be reduced [25]. To account for the relative contributions of movement behaviors to the overall time budget of the waking day, compositional data analysis (CoDA) should be applied [26–28]. CoDA has been increasingly used in studies of the associations of physical activity with health outcomes [29]. For example, in 415 Finnish men, time spent in moderate-vigorous intensity physical activity but not light physical activity was associated with cardiorespiratory fitness [30]. We employed CoDA to improve our understanding of a variety of physical activity intensities (including sitting) and different parameters of cardiorespiratory fitness derived during cardiopulmonary exercise testing, i.e., VO_2_ at rest, VO_2_VT1, relative VO_2_VT1, VO_2_peak, VO_2_ recovery 60 s, %predicted VO_2_peak, change in VO_2_ with increasing work (VO_2_/work), O_2_pulse, and maximum heart rate, in a population-based cross-sectional study from Northeast Germany. In addition to the VO₂peak, the additional markers provide valuable insights into baseline metabolism, submaximal efficiency, recovery kinetics, cardiorespiratory fitness relative to predicted norms, and the body’s ability to adapt to increasing physiological demands, offering a more comprehensive assessment of aerobic capacity and functional performance. Given the sex specific systemic effects of physical activity and exercise [31], we decided to stratify our models by sex. We hypothesize that most parameters are influenced only by moderate-vigorous physical activity (positive) and stationary time (inverse) but to a lesser degree by light physical activity. In particular, we investigated sex-specific differences that we suspect to be due to different adaptation processes to physical activity and thereby demonstrated the importance of individualized prescriptions of physical activity [31].

## Methods

### Study design and population

Our analysis was conducted with data from the Study of Health in Pomerania (SHIP). An overview of the entire study and recruitment details has been published elsewhere [32]. In brief, a random cluster sample (age range 20 to 79 years) was drawn from the population of West Pomerania, the north-eastern region of Germany. The net sample (without migrated or deceased persons) comprised 6,265 eligible individuals with 4,308 (2,193 women) of them participating in the baseline (SHIP-START-0) study (response 68.8%). All subjects who participated in the baseline SHIP were re-invited to take part in the first examination follow-up (SHIP-START-1), which was realized from 2002 to 2006. Of the 3,949 persons eligible for SHIP-START-1, 3,300 subjects were re-examined, resulting in a follow-up response of 83.6%. For the second examination follow-up (SHIP-START-2)18,19 conducted from 2008 to 2012, all 3,708 eligible individuals that participated in the baseline study were re-invited. Of them, 2,333 were re-examined (follow-up response of 62.9%).

**Fig. 1:**
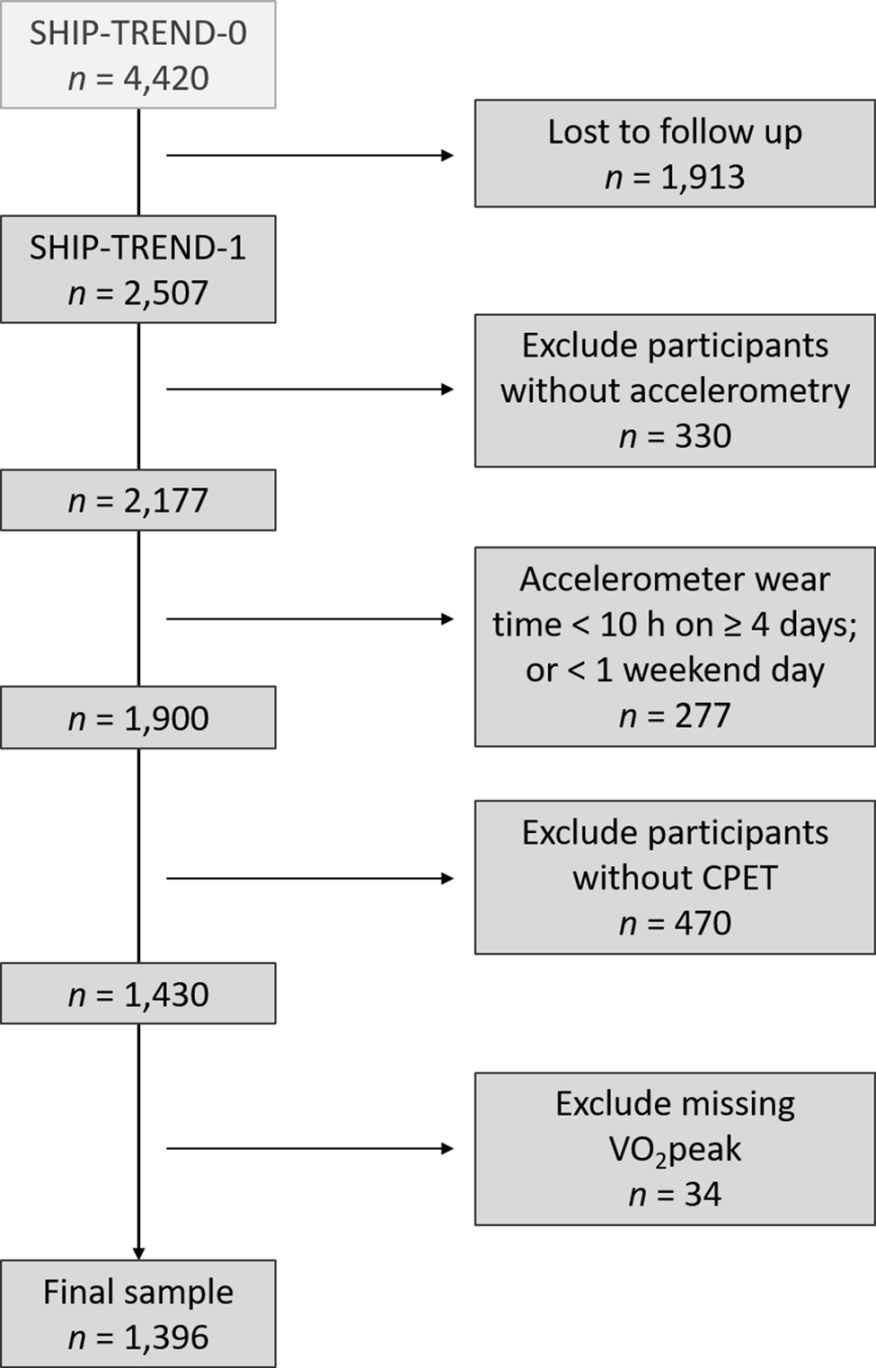
Flow chart of the analysis sample. CPET = Cardiopulmonary exercise testing. VO_2_peak = peak oxygen uptake

While SHIP-START-2 was being conducted, between 2008 and 2012, a second independent cohort was established, called SHIP-TREND-018,19, covering the same region as the initial SHIP. A stratified random sample of 8,826 adults, aged 20 to 79 years, was selected. Participation in the initial SHIP cohort was an exclusion criterion. In total, 4,420 individuals participated in SHIP-TREND-0 (response 50.1%). For the present study, we performed cross-sectional analyses using pooled data from SHIP-START-2 and SHIP-TREND-0 (*n* = 6,753 individuals; 3,510 women [52.0%]). SHIP-TREND-1 is the first follow-up examination of SHIP-TREND-0 (IRB approval number BB 39/08). For the follow-up, 2,507 adults participated between 2016 and 2019. The study was approved by the local ethics committee and was in accordance with the Declaration of Helsinki (IRB approval number BB 174/15). All study participants signed informed consent forms to participate in this study. This specific project has been approved under SHIP/2023/06/D. The participants provided a written declaration of consent before their study examination. A total of 1,900 individuals provided valid accelerometer data (wear time criteria: ≥ 10 h on ≥ 4 days, including ≥ 1 weekend day) [33]. Of those, 1,430 participants took part in the cardiopulmonary exercise testing. After excluding participants with missing VO_2_peak values, 1,396 remained in the sample for our analysis. The flow of participation is shown in Fig. 1.

### Interview and medical examination

Certified interviewers took study participants through computer-assisted personal interviews. The collected data included sex, age, education, smoking status, partnership, and intake of medication. For education, the categories were less than, equal to, and more than 10 years of school attendance. Smoking status was grouped into current, former, and never smokers. Height and weight, as well as waist and hip circumference, were acquired via standardized anthroprometric measurements. Body mass index (BMI) was calculated by dividing weight in kg by height in m squared.

### Cardiopulmonary exercise test

The cardiopulmonary exercise test parameters were obtained via a modified Jones protocol on an electromagnetic bicycle ergometer (Ergoselect 100, Ergoline, Germany). After 3 min of rest and 1 min of unloaded work (20 W), the workload increased by 16 W every minute. The tests were conducted at a controlled room temperature while blood pressure, oxygen saturation, and 12-lead ECG data were continuously monitored. Testing was terminated due to exhaustion of the participant (all RER > 1.05).

### Gas exchange variables

Starting with a calibration prior to cardiopulmonary exercise testing, gas exchange and ventilation were measured breath-by-breath via an Oxycon Pro with a Rudolph mask (JÄGER/VIASYS Healthcare System, Hoechberg, Germany). The values for oxygen uptake, tidal volume and carbon dioxide uptake were averaged over 10 s intervals. VO_2_peak values were defined as the highest values averaged over 10 s intervals at the maximum workload or in the early recovery stage. Resting VO_2_ was measured at rest. VO_2_/work was calculated by subtracting the resting VO_2_ from the VO_2_peak and then dividing it by the maximum load achieved. VO_2_VT1 was based on the V-slope method [34]. The relative VO_2_VT1 was calculated by subtracting VO_2_VT1 from VO_2_peak and then expressed as a percentage of VO_2_peak. The calculation of the %predicted VO_2_peak was described previously [35, 36]. Heart rate (HR) was measured via ECG during the CPET. The O2 pulse was calculated by dividing VO_2_peak by HRmax. VO_2_ recovery at 60 s was calculated by subtracting VO_2_ at 60 s after VO_2_peak from VO_2_peak and then expressed as a percentage of VO_2_peak. The values were normalized to the kg body weight of the participant. All the parameters are shown in Tables 1 and 2.

**Table 1:** Sample descriptions according to age-specific and sex-specific VO_2_peak quartiles for males (*n* = 706)

	VO_2_peak quartiles (IQR; mL/min/kg)				
	I 17.6–23.2	II 22.6–28.6	III 26.2–32.9	IV 31.0-40.5	*P* for trend
***n***	182	180	170	174	
**Age**,** years**	59.0 ± 13.4	58.6 ± 12.9	58.3 ± 13.3	58.1 ± 13.4	0.55
**School education**					< 0.01
< 10 years	23(13%)	15(8%)	21(12%)	15(9%)	
10 years	102(56%)	103(57%)	84(50%)	79(46%)	
> 10 years	56(31%)	62(34%)	65(38%)	79(46%)	
**Smoking status**					< 0.01
Current	44(24%)	26(14%)	23(14%)	9(5%)	
Former	112(62%)	110(61%)	91(54%)	97(56%)	
Never	26(14%)	44(24%)	56(33%)	68(39%)	
**Partnership**,** yes**	153(84%)	168(93%)	149(88%)	155(89%)	0.36
**BMI**,** kg/m**^**2**^	31.3 ± 5.1	29.4 ± 3.6	27.6 ± 3.2	25.6 ± 2.6	< 0.01
**Beta blocker usage**,** yes**	78(43%)	40(22%)	27(16%)	15(9%)	< 0.01
**Accelerometry**					
Wear time, hours day^− 1^	14.1 ± 1.6	14.5 ± 1.5	14.6 ± 1.7	14.7 ± 1.4	< 0.01
MVPA, hours day^− 1^	0.6 ± 0.4	0.7 ± 0.4	0.8 ± 0.6	1.0 ± 0.5	< 0.01
LPA, hours day^− 1^	3.1 ± 0.9	3.6 ± 1.0	3.3 ± 0.9	3.5 ± 0.9	< 0.01
ST, hours day^− 1^	10.4 ± 1.6	10.4 ± 1.5	10.4 ± 1.7	10.2 ± 1.6	0.41
MVPA as percent of wear time, %	4.5 ± 2.9	5.0 ± 2.8	5.7 ± 3.6	6.6 ± 3.4	< 0.01
LPA as percent of wear time, %	21.6 ± 6.0	23.2 ± 6.2	22.8 ± 6.1	24.0 ± 6.1	< 0.01
ST as percent of wear time, %	73.9 ± 7.6	71.8 ± 7.8	71.5 ± 7.7	69.4 ± 7.9	< 0.01
**CPET**					
Resting VO_2_, mL/min/kg	3.9 ± 1.5	3.9 ± 1.4	4.4 ± 1.6	4.7 ± 2.0	< 0.01
VO_2_/work, mL/watt	0.1 ± 0.02	0.1 ± 0.02	0.1 ± 0.02	0.1 ± 0.02	< 0.01
VO_2_VT1, mL/min/kg	11.8 ± 2.4	13.5 ± 2.6	15.4 ± 3.2	18.5 ± 5.1	< 0.01
Relative VO_2_VT1, difference as % of VO_2_peak	41.8 ± 10.5	47.5 ± 10.6	47.6 ± 10.3	48.4 ± 10.3	< 0.01
VO_2_peak, mL/min/kg	20.6 ± 4.4	26.0 ± 3.9	29.7 ± 4.8	36.1 ± 6.6	< 0.01
%predicted VO_2_peak	84.9 ± 13.1	102.4 ± 11.3	110.5 ± 10.8	126.6 ± 14.6	< 0.01
HRmax (beats/min)	128.2 ± 27.9	148.6 ± 22.5	153.9 ± 23.1	158.7 ± 18.8	< 0.01
O_2_pulse, mL/beat	16.1 ± 4.4	16.4 ± 3.2	16.7 ± 3.0	18.0 ± 3.0	< 0.01
VO_2_ recovery 60 s, difference as % of VO_2_peak	36.2 ± 11.3	41.1 ± 9.7	44.9 ± 9.6	47.6 ± 8.2	< 0.01

BMI, body mass index; CPET, cardiopulmonary exercise; HRmax, maximal heart rate; LPA, light physical activity; MVPA, moderate-to-vigorous physical activity; ST, sedentary time; VO_2_VT1, maximal oxygen consumption at the anaerobic threshold

The values are presented as mean ± standard deviation for continuous variables. For categorical variables, numbers and percentages are provided

**Table 2:** Sample descriptions according to age-specific and sex-specific VO_2_peak quartiles for fem**ales (*n*** **= 690)**

	VO_2_peak quartiles (IQR; mL/min/kg)				
	I 16.3–19.7	II 20.2–24.4	III 23.0-27.5	IV 27.5–33.4	*P* for trend
***n***	181	171	170	168	
**Age**,** years**	56.7 ± 13.2	55.5 ± 13.2	55.2 ± 12.7	55.4 ± 12.5	0.26
**School education**					< 0.01
< 10 years	25(14%)	15(9%)	13(8%)	6(4%)	
10 years	117(66%)	106(62%)	91(54%)	92(55%)	
> 10 years	39(22%)	50(29%)	66(39%)	70(42%)	
**Smoking status**					0.51
Current	32(18%)	23(13%)	31(18%)	25(15%)	
Former	79(44%)	64(37%)	76(45%)	65(39%)	
Never	70(39%)	84(49%)	63(37%)	78(46%)	
**Partnership**,** yes**	152(84%)	143(84%)	133(78%)	132(79%)	0.10
**BMI**,** kg/m**^**2**^	31.6 ± 5.2	27.5 ± 4.5	26.1 ± 3.8	24.1 ± 3.4	< 0.01
**Beta blocker**,** yes**	57(31%)	45(26%)	23(14%)	20(12%)	< 0.01
**Accelerometry**					
Wear time, hours day^− 1^	13.9 ± 1.4	14.2 ± 1.1	14.5 ± 1.6	14.5 ± 1.3	< 0.01
MVPA, hours day^− 1^	0.6 ± 0.3	0.7 ± 0.4	0.7 ± 0.3	0.8 ± 0.3	< 0.01
LPA, hours day^− 1^	3.5 ± 1.0	3.6 ± 1.0	3.6 ± 0.8	3.7 ± 1.0	0.02
ST, hours day^− 1^	9.9 ± 1.4	9.9 ± 1.3	10.2 ± 1.7	10.0 ± 1.3	0.24
MVPA as percent of wear time, %	4.1 ± 2.4	4.6 ± 2.7	4.7 ± 2.4	5.2 ± 2.4	< 0.01
LPA as percent of wear time, %	25.0 ± 6.3	25.5 ± 6.4	24.8 ± 5.9	25.7 ± 6.1	0.42
ST as percent of wear time, %	70.9 ± 7.5	69.9 ± 7.3	70.5 ± 7.0	69.0 ± 7.0	0.02
**CPET**					
Resting VO_2_, mL/min/kg	3.4 ± 1.3	3.8 ± 1.6	4.3 ± 2.0	4.5 ± 1.8	< 0.01
VO_2_/work, mL/watt	0.1 ± 0.03	0.1 ± 0.02	0.2 ± 0.03	0.2 ± 0.03	< 0.01
VO_2_VT1, mL/min/kg	11.0 ± 1.8	12.8 ± 2.3	13.9 ± 2.5	17.2 ± 3.4	< 0.01
Relative VO_2_VT1, difference as % of VO_2_peak	38.2 ± 10.3	42.1 ± 9.3	44.5 ± 9.8	43.7 ± 9.6	< 0.01
VO_2_peak, mL/min/kg	18.0 ± 2.6	22.2 ± 2.6	25.3 ± 3.0	30.7 ± 4.7	< 0.01
%predicted VO_2_peak	100.8 ± 12.8	111.4 ± 14.8	121.4 ± 13.7	139.4 ± 17.9	< 0.01
HRmax (beats/min)	136.3 ± 24.5	147.3 ± 21.3	152.7 ± 20.9	155.7 ± 19.6	< 0.01
O_2_pulse, mL/beat	11.5 ± 2.1	11.4 ± 2.1	11.8 ± 2.1	12.9 ± 2.2	< 0.01
VO_2_ recovery 60 s, difference as % of VO_2_peak	32.7 ± 9.7	36.4 ± 8.1	38.8 ± 9.0	42.5 ± 8.5	< 0.01

BMI, body mass index; CPET, cardiopulmonary exercise; HRmax, maximal heart rate; LPA, light physical activity; MVPA, moderate-to-vigorous physical activity; ST, sedentary time; VO_2_VT1, maximal oxygen consumption at the anaerobic threshold

The values are presented as mean ± standard deviation for continuous variables. For categorical variables, numbers and percentages are provided

### Movement behaviors

Data on physical activity and stationary time were gathered via triaxial ActiGraph Model GT3X + accelerometers (Pensacola, FL). Using ActiLife version 6.13.3 (ActiGraph, Pensacola, FL), the accelerometers were initialized at a sampling rate of 30 Hz, and the raw data were integrated into 10 s epochs. The accelerometer was worn for a period of 7 days on an elastic belt on the right side of the hip. The participants were instructed to wear the accelerometer throughout the whole waking day, excluding night sleep and activities involving water, such as showering. ActiGraph accelerometers measure counts per minute (cpm). For the present analysis, counts of the vertical axis were used (representative of the steps). To identify the accelerometer wear time as well as the time spent with different physical activity intensities, cutoff points according to Troiano and colleagues were applied [37]. Wear time was determined by removing nonwear time, which was defined as at least 60 min of consecutive zero counts, allowing for 2 min of counts between 0 and 100. The time spent in moderate-vigorous physical activity was determined by summing minutes per day where the accelerometer count met the intensity threshold criterion of 2020 cpm (i.e., activities of 3 METs or more, such as brisk walking). Light physical activity was defined as 100–2019 cpm. A time of less than 100 cpm was defined as stationary time [37].

Time spent physically active or stationary are compositional components of total accelerometer wear time. These variables were expressed as proportions of total time and then isometric log-ratio transformed [27, 38] to the following z parameters which were used as exposure variables in the analyses.

1 $$\:z1\:=\:\surd\:\frac{2}{3}\text{l}\text{n}\frac{sedentary\:time}{\sqrt{LPA\:\text{x}\:MVPA}}$$

2 $$\:z2\:=\:\surd\:\frac{1}{2}\text{l}\text{n}\frac{LPA}{MVPA}$$

By entering the isometric-log-transformed variables (z1 and z2) into the model the combined effect of proportions of time spent in the three different movement behaviors (stationary, light physical activity and moderate-to-vigorous physical activity) and therefore of the entire composition is taken into account.

### Statistical analysis

Age-specific VO_2_peak quartiles were used to describe the male and female study population, respectively. For the age-specific quartiles, we stratified the study population by 10-year age strata and calculated the quartiles for each stratum. *P* for trend was calculated to compare differences across VO_2_peak quartiles. The compositional mean is a better representative of the center of a cloud of compositional data points than the usual arithmetic mean [24, 25]. The compositional mean was obtained by computing the geometric mean for each behavior separately and then normalizing the data to 100% [24, 25]. Sex-specific linear regression models were used to examine the associations of moderate-vigorous physical activity, light physical activity, and stationary time with parameters gathered during cardiopulmonary exercise testing. The entire composition of the daily time spent in the 3 behaviors acts as an exposure variable by entering the *z* parameters in the models. All models were adjusted for age, education, smoking, and partnership, except the %predicted VO_2_peak model, where age was omitted, as it is part of the calculation of the %predicted VO_2_peak. In models examining O_2_pulse or HRmax, individuals using beta blockers were excluded. The normality and homoscedasticity of the residuals were assessed via kernel density plots, Q‒Q plots, and residual‒vs.-fitted plots. *P* values < 0.05 were considered statistically significant. The 95% confidence intervals are omitted because they are meaningless in a compositional paradigm [24, 25]. Sensitivity analysis was conducted for females before or after menopause. Menopause status was assessed with a questionnaire regarding the absence of menorrhoea. All analyses were conducted using Stata version 15.1 and R version 4.1.3.

## Results

### Study population

**Fig. 2:**
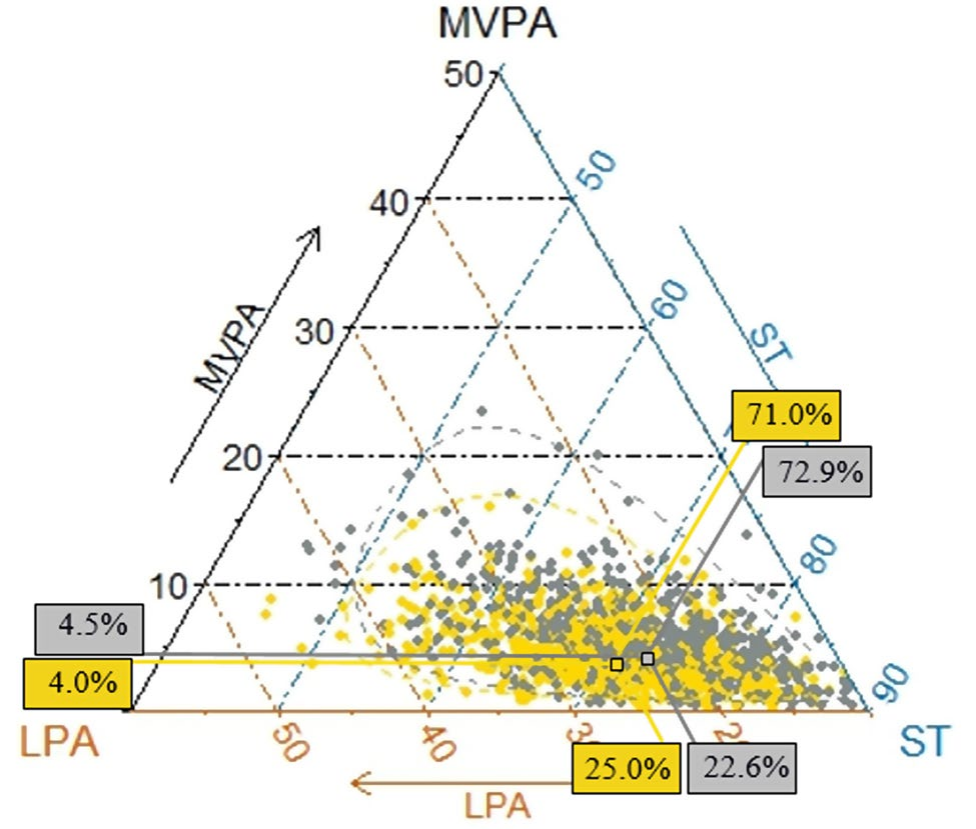
Ternary plot of the sample composition of time spent in moderate-to-vigorous physical activity (MVPA), light physical activity (LPA), and sedentary time (ST) (all in % of 100% accelerometer wear time) with means (squares) and 95% confidence intervals (dashed lines), separately for males (gray) and females (yellow)

A total of 1,396 participants with a mean age of 57.1 (SD 13.2, 51% men) years were included in the analysis. The sample characteristics stratified by age- and sex-specific VO_2_peak quartiles for males and females are presented in Tables 1 and 2, respectively. Participants with higher VO_2_peak values had significantly higher levels of education, a lower BMI, and a lower proportion of participants who took beta-blockers. A higher VO_2_peak was associated with a lower prevalence of smoking in males but not females. In both sexes, a greater VO_2_peak was related to a greater accelerometer wear time, higher absolute and relative moderate-vigorous and light physical activity. While the relative stationary time was lower in study participants with higher VO_2_peak, absolute stationary time showed no differences between quartiles The distribution of the compositional data is shown in ternary plots with truncated plot axes according to the distribution of the data for better readability (see supplementary Fig. S1). The mean percentages of time spent in moderate-vigorous and light physical activity as well as stationary time were 4.5%, 22.6%, and 72.9%, respectively, in males and 4.0%, 25.0%, and 71.0% in females, respectively (Fig. 2).

### Associations between movement behavior compositions and CPET parameters

The results of the linear regression models for both sexes are presented in Table 3. The relative distribution of time among the three movement behaviors as a whole (including the adjusted confounders) was significantly associated with all parameters of cardiopulmonary exercise testing. The amount of variance explained by the models varied across outcomes and was generally greater in males than in females, except for resting VO_2_ (R^2^_adj_ = 0.02 and 0.04, respectively) and HRmax (R^2^_adj_ = 0.34 and 0.38, respectively). In males, the proportion of time spent in the stationary time was inversely associated with VO_2_/work (b = -0.01, *p* = 0.02), and this was the only part of the composition significantly associated with this outcome. In contrast, the proportion of time spent in light physical activity was inversely related to VO_2_/work, and the proportion of time spent in moderate-vigorous physical activity was positively related to VO_2_/work in females (b = -0.01, *p* = 0.04 and b = 0.01, *p* < 0.01, respectively). Time spent in moderate-vigorous physical activity was positively associated with VO_2_VT1 in both males (b = 1.18, *p* < 0.01) and females (b = 1.08, *p* < 0.01), whereas in males, only stationary time was inversely related to VO_2_VT1 (b = -2.00, *p* < 0.01). Similarly, moderate-vigorous physical activity was positively associated with VO_2_peak in both males (b = 2.59, *p* < 0.01) and females (b = 1.79, *p* < 0.01). Only in males was the stationary time inversely related to the VO_2_peak (b = -3.22, *p* < 0.01). In both males and females, higher light physical activity (b = 6.48, *p* = 0.02 and b = 7.44, *p* = 0.02, respectively) and lower stationary time (b = -11.62, *p* < 0.01 and b = -7.86, *p* < 0.01, respectively) were related to higher %predicted VO_2_peak. In males, greater moderate-vigorous physical activity was also related to a greater %predicted VO_2_peak (b = 5.14, *p* < 0.01). For HRmax, only moderate-vigorous physical activity in males was significantly associated (b = 4.87, *p* < 0.01). In females, the stationary time was inversely related to the O2pulse (b = -0.74, *p* = 0.02). In both sexes, moderate-vigorous physical activity was positively related to VO_2_ recovery at 60 s (b = 2.42, *p* < 0.01 in men and b = 2.68, *p* < 0.01 in females). Only in females was stationary time inversely associated with this outcome (b = -3.07, *p* < 0.01). No parts of the composition were significantly associated with resting VO_2_ or relative VO_2_VT1, neither in males nor in females. The results of the sensitivity analysis among females according to menopausal status are shown in supplementary table S1.

**Table 3:** Compositional behavior models for CPET parameters for the proportion of the day spent in each movement behavior: moderate-to-vigorous physical activity (MVPA), light physical activity (LPA), and sedentary time (ST), separately for men and women

CPET parameter	MVPA		LPA		ST		Composition		
	y_1_^1^	*p*	y_1_^2^	*p*	y_1_^3^	*p*	*n*	adj. *R*^2^	*p*
Males									
Resting VO_2_	0.20	0.13	0.15	0.54	-0.35	0.09	704	0.02	**0.01**
VO_2_/work	0.00	0.15	0.00	0.27	**-0.01**	**0.02**	700	0.03	**< 0.01**
VO_2_VT1	**1.18**	**< 0.01**	0.82	0.18	**-2.00**	**< 0.01**	700	0.15	**< 0.01**
Relative VO_2_VT1	1.51	0.06	-1.80	0.23	0.30	0.81	700	0.15	**< 0.01**
VO_2_peak	**2.59**	**< 0.01**	0.64	0.46	**-3.22**	**< 0.01**	704	0.43	**< 0.01**
%predicted VO_2_peak	**5.14**	**< 0.01**	**6.48**	**0.02**	**-11.62**	**< 0.01**	701	0.14	**< 0.01**
HRmax	**4.87**	**< 0.01**	-5.73	0.06	0.86	0.73	543	0.34	**< 0.01**
O_2_pulse	0.17	0.59	0.51	0.38	-0.69	0.15	543	0.09	**< 0.01**
VO_2_ recovery 60 s	**2.42**	**< 0.01**	-1.35	0.32	-1.08	0.33	703	0.32	**< 0.01**
Females									
Resting VO_2_	0.13	0.38	-0.16	0.56	0.03	0.89	690	0.04	**< 0.01**
VO_2_/work	**0.01**	**< 0.01**	**-0.01**	**0.04**	0.00	0.47	688	0.01	**0.02**
VO_2_VT1	**1.08**	**< 0.01**	-0.43	0.41	-0.66	0.14	687	0.09	**< 0.01**
Relative VO_2_VT1	0.01	1.00	0.26	0.86	-0.26	0.84	687	0.13	**< 0.01**
VO_2_peak	**1.79**	**< 0.01**	-0.60	0.44	-1.19	0.07	690	0.28	**< 0.01**
%predicted VO_2_peak	0.42	0.79	**7.44**	**0.02**	**-7.86**	**< 0.01**	689	0.07	**< 0.01**
HRmax	1.54	0.32	-0.66	0.81	-0.88	0.70	545	0.38	**< 0.01**
O_2_pulse	0.19	0.35	0.54	0.14	**-0.74**	**0.02**	545	0.04	**< 0.01**
VO_2_ recovery 60 s	**2.68**	**< 0.01**	0.39	0.76	**-3.07**	**< 0.01**	689	0.29	**< 0.01**

Statistically significant associations at the 95% confidence level (*p* < 0.05) are highlighted in bold. The regression coefficient corresponds to the change in the log ratio of the given behavior to the others. The 95% confidence intervals are omitted, as they are meaningless in a compositional paradigm. The models were adjusted for age, education, smoking, and partnership, except in the %predicted VO_2_peak model; age was omitted, as it is part of the computation of the %predicted VO_2_peak. In the models for HRmax and O2pulse, individuals using medications for high blood pressure were excluded

### Mapping the effects of the behavior compositions on the parameters of the cardiopulmonary exercise test

**Fig. 3:**
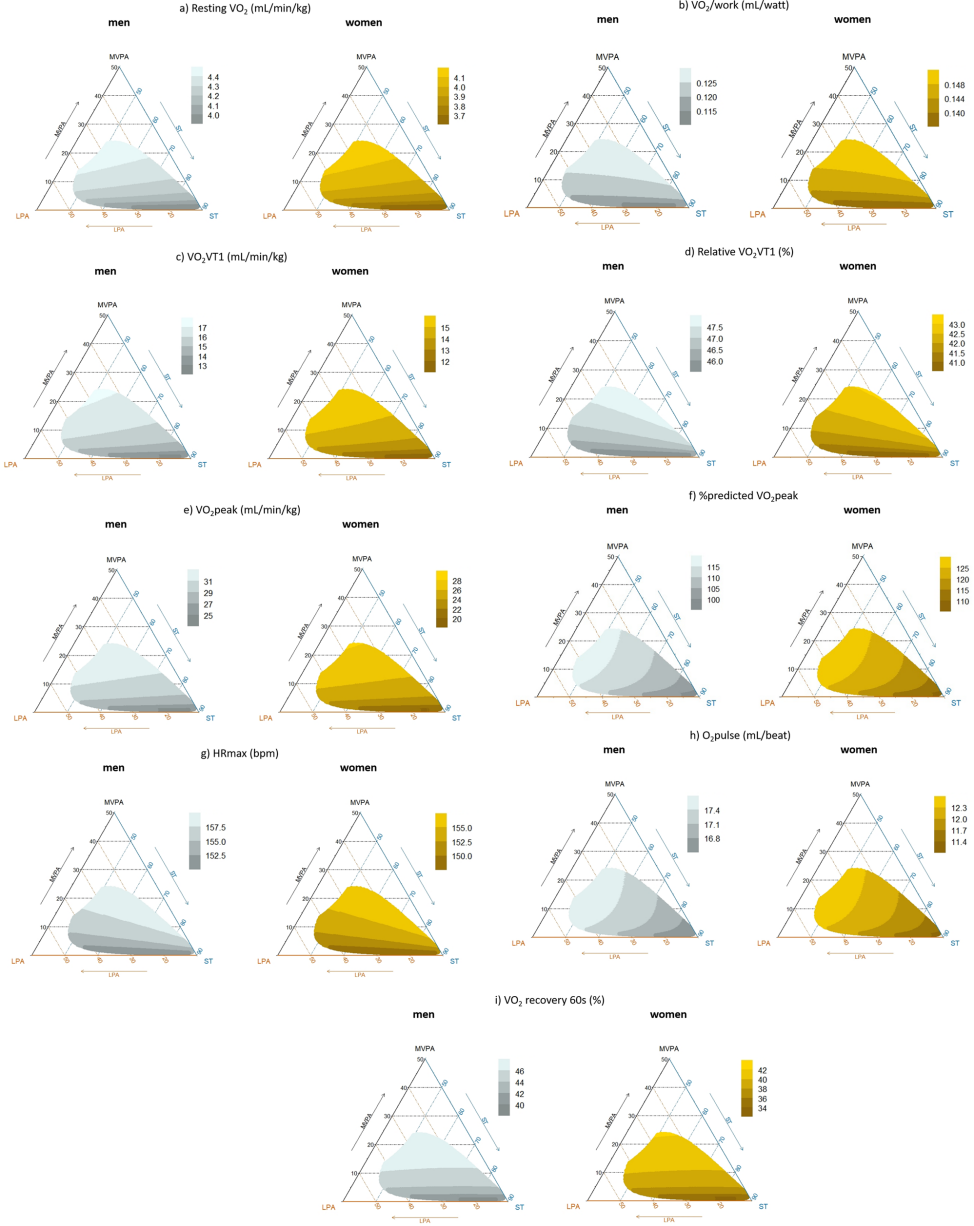
Estimated (**a**) resting VO_2_, (**b**) VO_2_/work, (**c**) VO_2_VT1, (**d**) relative VO_2_VT1, (**e**) VO_2_peak, (**f**) %predicted VO_2_peak, (**g**) HRmax, (**h**) O_2_pulse, and (**i**) VO_2_ recovery 60 s, as a function of the proportion of time spent in moderate-to-vigorous physical activity (MVPA), light physical activity (LPA), and sedentary time (ST) (all in % of 100% accelerometer wear time) in males (gray, left) and females (yellow, right)

CoDA models were used to estimate the effects of different compositions of waking days (i.e., accelerometer wear time) on the parameters of the cardiopulmonary exercise test. These are presented for each outcome as a heatmap on a ternary plot showing the outcome for the composition of time spent in moderate-vigorous and light physical activity as well as stationary time during the waking day (total accelerometer wear time) (Fig. 3a-i). Estimations were computed separately for males and females, those aged 57 years (i.e., the mean age of the sample), never-smoker, with 10 years of school education, and those with a partner.

The plots show that moderate-vigorous physical activity positively affects all outcomes when either light physical activity or stationary time is replaced. However, the associations were less pronounced for %predicted VO_2_peak and O2pulse, whereas in both males and females, the outcome varied only for very low levels of moderate-vigorous physical activity (Fig. 3f and h). For all outcomes, the magnitude appeared to vary depending on the proportion of time in light physical activity and stationary time. The association was relatively stronger at lower proportions of moderate-vigorous physical activity and weakened as the proportion of time spent in moderate-vigorous physical activity increased. This also varied depending on the proportion of time in light physical activity as well as stationary time and outcomes of the cardiopulmonary exercise test.

The effects of light physical activity were less pronounced but appeared inverse when moderate-vigorous physical activity was replaced on any outcome. In turn, light physical activity had positive effects when stationary time was replaced, most notably on %predicted VO_2_peak and O_2_pulse (Fig. 3f and h). The proportion of time spent stationary had notable negative effects on all outcomes when moderate-vigorous physical activity was replaced. However, the magnitude of these associations appeared to depend on the relative proportion of light physical activity. The negative associations of stationary time were less pronounced when replaced by light physical activity and—in line with what was described above—were most noticeable in terms of %predicted VO_2_peak and O_2_pulse (Fig. 3f and h).

## Discussion

The aim of this study was to relate physical activity intensities (i.e. light and vigorous-to-moderate as well as stationary activity) with parameters derived from cardiopulmonary exercise testing. While VO_2_peak is used as the benchmark measurement for cardiorespiratory fitness, a variety of other parameters are measured during this test. Here, we assessed the relative contribution of these physical activity subgroups to VO_2_peak and report that the proportion of time spent in moderate-vigorous intensity as well as a reduction in the proportion of stationary time (for males only) were positively associated with VO_2_peak. A recent meta-analysis including 34 cohort studies emphasized the importance of increasing cardiorespiratory fitness to reduce all-cause mortality, cardiovascular disease and cancer [39]. A previous study reported that replacing time spent in other movement behaviors with vigorous physical activity was related to a greater VO_2_peak [40]. Interestingly, we found that the proportion of time spent in light intensity physical activity was not associated with VO_2_peak. In line with our findings, a study among 415 Finnish men revealed positive associations of moderate-vigorous but not light physical activity with CRF [30]. Importantly, while engaging in light intensity physical activity has no relationship with VO_2_peak, it can still have health benefits. A cross-sectional analysis of six studies with over 15,000 participants from the Prospective Physical Activity, Sitting and Sleep consortium revealed benefits from reallocating sedentary time to light physical activity regarding cardiometabolic outcomes [41]. A study including 2,223 NHANES participants revealed that an additional hour of sedentary time per day was associated with 0.12 and 0.24 MET decreases in cardiorespiratory fitness for males and females, respectively [42]. Overall, our results and those of previous studies suggest that moderator-vigorous intensity physical activity is essential for increasing VO_2_peak. Compared with previous studies, our results highlight the importance of sex with respect to the relationship between physical activity behavior and VO_2_peak.

In addition to the VO2peak, cardiopulmonary exercise testing provides further parameters that enable a more precise assessment of the performance of the cardiovascular, respiratory, muscular and metabolic systems. These markers may be useful for individual training and exercise control in healthy individuals or patients with cardiac or pulmonary diseases beyond VO_2_peak [14, 37]. For example, a previous study demonstrated that VO_2_VT1 significantly improved the prediction of long-term cardiovascular mortality in addition to VO_2_peak [43]. In patients with heart failure, the peak O2pulse can be used as an indicator of possible improvement in VO_2_peak through exercise training [44]. A low VO_2_/WR can be a sign of initial cardiac limitation to exercise, and flattening (loss of linearity) or downsloping can be visible in advanced heart failure [11]. Recently, Nayor and colleagues related the intensities of physical activity with cardiorespiratory fitness in 1,720 community-dwelling participants in the Framingham Heart Study [45]. Sedentary time, steps per day, and moderate-vigorous intensity physical activity were measured for 7 days with an accelerometer and were related to several cardiopulmonary exercise testing parameters. These movement behaviors were associated with parameters in early–moderate, late and recovery phases of the cardiopulmonary exercise test, with moderate-vigorous intensity physical activity resulting in the highest effect sizes [45]. With respect to our CoDA models, we can confirm the effect of the proportion of time spent in moderate-to-vigorous physical activity on parameters of the exercise and recovery phases of the cardiopulmonary exercise test and thus an extensive adaptation response triggered by physical activity with high-intensity.

Our results suggest significant differences in sex-specific relationships between movement behaviors and cardiorespiratory fitness. The proportion of time spent in activities with moderate-to-vigorous intensity was significantly associated with five parameters of the cardiopulmonary exercise test in males and females. Interestingly, four of these five parameters were matched for males and females (Table 3). In contrast, the proportion of sedentary time was significantly associated with four parameters of the cardiopulmonary exercise test in males and three in females. Only one parameter (%predicted VO_2_peak) was relevant in both sexes. The systemic response to a cardiopulmonary exercise test was differentially influenced by the proportional amounts of time spent being physically active or sedentary. Physiological differences between the sexes with respect to physical training response are based on a number of mechanisms. Differences in muscle strength, oxygen consumption, fiber type physiology, substrate metabolism, and muscle fatigue are explained by chromosomal and hormonal differences. Moreover, the epigenome and transcriptome play important roles at the molecular level in the response to exercise [46]. The influence of these mechanisms on cardiorespiratory fitness and the early, peak, and recovery parameters of the cardiopulmonary exercise test is likely and a potential explanation for our findings. Not considering these sex-specific differences in physiology and response to exercise may result in training programs that lead to suboptimal outcomes in both healthy populations and clinical patients [31]. We suggest that these sex-specific characteristics should be considered in future research and recommendations for the amount of time spent on different forms of physical activity in preventive and therapeutic settings.

There are also limitations of our study that should be considered. First, selection bias of highly motivated and physically active individuals is likely. Second, hip-worn accelerometers may not capture upper body activities such as lifting or carrying weight. Accelerometers must be removed during activities in water [47]. Furthermore, these devices cannot discriminate between sitting and standing motionless [48]. Third, the study included mainly Caucasians. Whether our findings are applicable to other ethnicities is unclear. Fourth, the cross-sectional study design allows no evaluation of cause and effect. Fifth, sex-specific effects may be driven by the number of statistical tests and need to be confirmed by larger studies. Finally, additional residual confounding by determinants that affect movement behavior and cardiorespiratory fitness cannot be excluded.

The strengths of this study are the comparatively large sample size and the highly standardized data acquisition process. Furthermore, the use of CoDA accounts for the relative nature of time-based movement behaviors and offers an evaluation that is more precise and insightful than former approaches of examining each behavior separately.

## Conclusion

Relative amounts of accelerometer-based time spent in moderate to vigorous and light physical activity as well as sedentary time showed different associations in males and females with multiple parameters of cardiopulmonary exercise testing. Our results support previous evidence by revealing positive relationships between moderate-to-vigorous physical activity and the majority of investigated cardiopulmonary exercise testing parameters and similar relationships in males and females. Light intensity physical activity seems to be less relevant. Sedentary time is important for almost as many fitness parameters but inverse compared to moderate to vigorous physical activity. Albeit, we identified differences with regards to the relationship between stationary time and cardiorespiratory fitness, the effects were rather small. Hence, reducing low physical activity levels for health promotion should be a priority irrespective of sex.

## Electronic supplementary material

Below is the link to the electronic supplementary material.

Supplementary Material 1

Supplementary Material 2

Supplementary Material 3

## Data Availability

The data of the SHIP participants analyzed during this study cannot be made publically available owing to the informed consent of the participants. It can be accessed through an application form at https://transfer.ship-med.uni-greifswald.de/FAIRequest/ for researchers who meet the criteria for access to confidential data.
