# Supplementary material for: Movement behaviors and cardiorespiratory fitness – a cross-sectional compositional data analysis among German adults
Source: BMC Sports Sci Med Rehabil. 2025 Mar 28;17:63. doi: 10.1186/s13102-025-01112-7 (PMC11951759; doi:10.1186/s13102-025-01112-7)
Supplement: Supplementary file 1 — Supplementary Material 1 [file 13102_2025_1112_MOESM1_ESM.docx]

**Figure S1** Ternary plots of the total sample composition of time spent in moderate-to-vigorous physical activity (MVPA), light physical activity (LPA), and sedentary time (ST) (all in % of 100% accelerometer wear time). The plot on the right is zoomed-in from the plot on the left for better readability with the MVPA axis ranging from 0 to 50%, the LPA axis ranging from 10 to 60%, and the ST axis ranging from 40 to 90%.
