# Supplementary figures and images for: Movement behaviors and cardiorespiratory fitness – a cross-sectional compositional data analysis among German adults
Source: BMC Sports Sci Med Rehabil. 2025 Mar 28;17:63. doi: 10.1186/s13102-025-01112-7 (PMC11951759; doi:10.1186/s13102-025-01112-7)

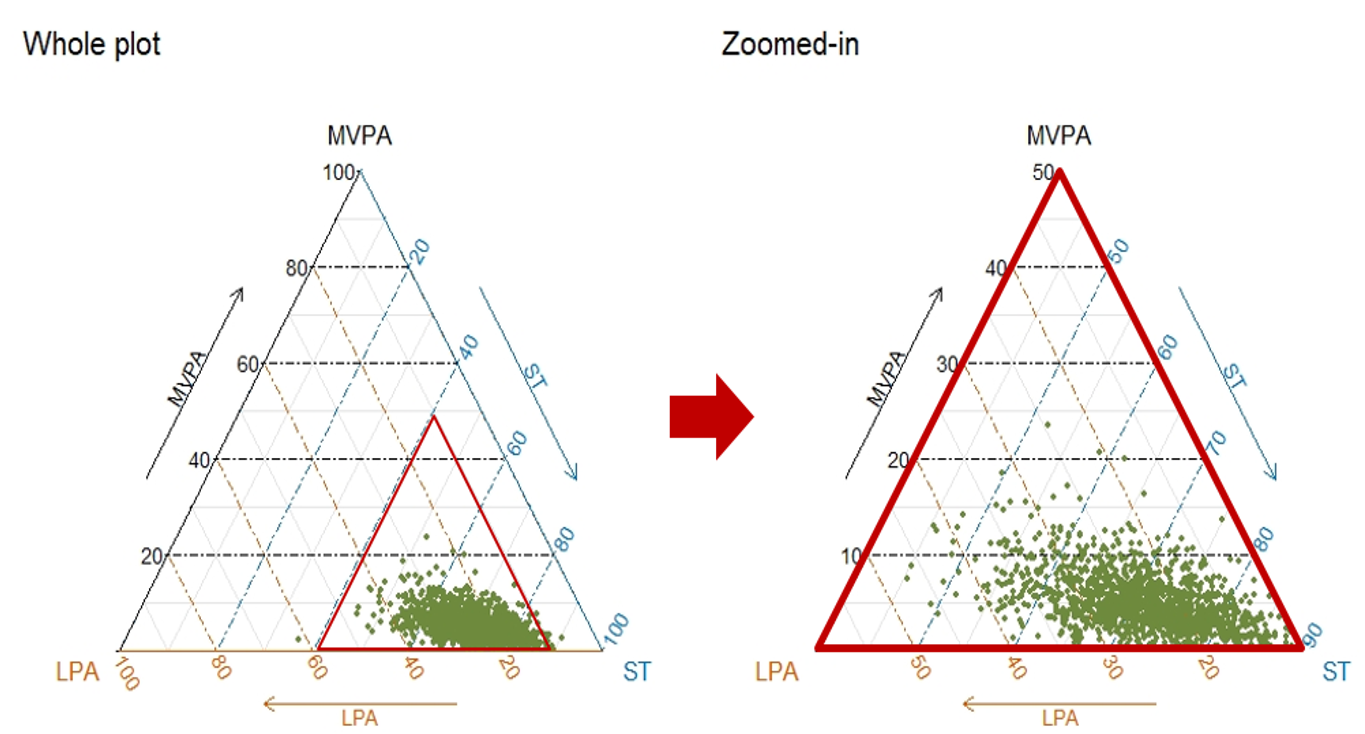

Supplement: Supplementary file 2 — Supplementary Material 2 [file 13102_2025_1112_MOESM2_ESM.tif]
