# Supplementary material for: Movement behaviors and cardiorespiratory fitness – a cross-sectional compositional data analysis among German adults
Source: BMC Sports Sci Med Rehabil. 2025 Mar 28;17:63. doi: 10.1186/s13102-025-01112-7 (PMC11951759; doi:10.1186/s13102-025-01112-7)
Supplement: Supplementary file 3 — Supplementary Material 3 [file 13102_2025_1112_MOESM3_ESM.docx]

**Table S1** Compositional behavior models for CPET parameters for proportion of the day spent in each movement behavior; moderate-to-vigorous physical activity (MVPA), light physical activity (LPA), and sedentary time (ST), separately for pre- and post-menopausal women.

| CPET parameter | MVPA | | LPA | | ST | | Composition | | |
| --- | --- | --- | --- | --- | --- | --- | --- | --- | --- |
|  | y_1_^1^ | p | y_1_^2^ | p | y_1_^3^ | p | n | adj. R^2^ | p |
| Pre-menopausal |  |  |  |  |  |  |  |  |  |
| Resting VO_2_ | 0.28 | 0.13 | -0.18 | 0.74 | -0.10 | 0.81 | 204 | 0.02 | 0.15 |
| VO_2_/work | **0.01** | **0.04** | -0.01 | 0.09 | 0.01 | 0.53 | 203 | 0.01 | 0.25 |
| VO_2_VT1 | **2.31** | **<0.01** | **-2.67** | **0.02** | 0.35 | 0.70 | 204 | 0.15 | **0.04** |
| Relative VO_2_VT1 | -0.99 | 0.54 | 1.46 | 0.60 | -0.46 | 0.84 | 204 | 0.04 | **0.03** |
| VO_2_peak | **3.55** | **<0.01** | **-4.01** | **0.02** | 0.46 | 0.74 | 204 | 0.13 | **<0.01** |
| %predicted VO_2_peak | 5.82 | 0.09 | -1.72 | 0.77 | -4.10 | 0.41 | 204 | 0.04 | **0.02** |
| HRmax | -0.75 | 0.76 | 1.06 | 0.80 | -0.31 | 0.93 | 187 | 0.23 | **<0.01** |
| O_2_pulse | 0.51 | 0.23 | -0.11 | 0.87 | -0.40 | 0.51 | 187 | 0.05 | **0.01** |
| VO_2_ recovery 60s | **4.03** | **<0.01** | -0.83 | 0.72 | -1.20 | 0.09 | 204 | 0.13 | **<0.01** |
| Post-menopausal |  |  |  |  |  |  |  |  |  |
| Resting VO_2_ | 0.08 | 0.62 | -0.10 | 0.74 | 0.02 | 0.93 | 486 | 0.01 | 0.26 |
| VO_2_/work | **0.01** | **0.02** | -0.01 | 0.10 | 0.00 | 0.57 | 485 | 0.02 | **0.01** |
| VO_2_VT1 | **0.78** | **<0.01** | 0.18 | 0.74 | -0.97 | 0.06 | 483 | 0.07 | **<0.01** |
| Relative VO_2_VT1 | 0.19 | 0.83 | -0.09 | 0.96 | -0.09 | 0.95 | 483 | 0.09 | **<0.01** |
| VO_2_peak | **1.38** | **<0.01** | 0.27 | 0.75 | **-1.65** | **0.02** | 486 | 0.21 | **<0.01** |
| %predicted VO_2_peak | 1.28 | 0.47 | 6.85 | 0.06 | **-8.13** | **0.01** | 485 | 0.08 | **<0.01** |
| HRmax | 2.26 | 0.24 | -0.59 | 0.86 | -1.67 | 0.57 | 358 | 0.30 | **<0.01** |
| O_2_pulse | 0.10 | 0.66 | 0.60 | 0.15 | -0.71 | 0.05 | 358 | 0.04 | **<0.01** |
| VO_2_ recovery 60s | **2.29** | **<0.01** | 0.46 | 0.76 | **-2.75** | **0.04** | 485 | 0.24 | **<0.01** |

Statistically significant associations at the 95% confidence level (p < 0.05) are highlighted in bold. The regression coefficient corresponds to the change in the log-ratio of the given behavior to the others. The 95% confidence intervals are omitted, as they are meaningless in a compositional paradigm. The models were adjusted for age, education, smoking, and partnership, except in the %predicted VO_2_peak model, age was omitted as it is part of the computation of %predicted VO_2_peak. In models for HRmax and O_2_pulse, individuals using medications for high blood pressure were excluded
